# Impact of breast density on diagnostic accuracy in digital breast tomosynthesis versus digital mammography: results from a European screening trial

**DOI:** 10.1186/s13058-023-01712-6

**Published:** 2023-10-04

**Authors:** Jakob Olinder, Kristin Johnson, Anna Åkesson, Daniel Förnvik, Sophia Zackrisson

**Affiliations:** 1 Department of Translational Medicine, Radiology Diagnostics, Lund University, Skåne University Hospital, Carl-Bertil Laurells Gata 9, 20502 Malmö, Sweden; 2 Department of Imaging and Physiology, Skåne University Hospital, Malmö, Sweden; 3 Clinical Studies Sweden-Forum South, Skåne University Hospital, Lund, Sweden; 4 Department of Translational Medicine, Medical Radiation Physics, Lund University, Skåne University Hospital, Malmö, Sweden; 5 Radiation Physics, Department of Hematology, Oncology and Radiation Physics, Skåne University Hospital, Lund, Sweden

**Keywords:** Breast neoplasms, Early detection of cancer, Mammography, Logistic models

## Abstract

**Background:**

The diagnostic accuracy of digital breast tomosynthesis (DBT) and digital mammography (DM) in breast cancer screening may vary per breast density subgroup. The purpose of this study was to evaluate which women, based on automatically assessed breast density subgroups, have the greatest benefit of DBT compared with DM in the prospective Malmö Breast Tomosynthesis Screening Trial.

**Materials and methods:**

The prospective European, Malmö Breast Tomosynthesis Screening Trial (*n* = 14,848, Jan. 27, 2010–Feb. 13, 2015) compared one-view DBT and two-view DM, with consensus meeting before recall. Breast density was assessed in this secondary analysis with the automatic software Laboratory for Individualized Breast Radiodensity Assessment. DBT and DM’s diagnostic accuracies were compared by breast density quintiles of breast percent density (PD) and absolute dense area (DA) with confidence intervals (CI) and McNemar’s test. The association between breast density and cancer detection was analyzed with logistic regression, adjusted for ages < 55 and ≥ 55 years and previous screening participation.

**Results:**

In total, 14,730 women (median age: 58 years; inter-quartile range = 16) were included in the analysis. Sensitivity was higher and specificity lower for DBT compared with DM for all density subgroups. The highest breast PD quintile showed the largest difference in sensitivity and specificity at 81.1% (95% CI 65.8–90.5) versus 43.2% (95% CI 28.7–59.1), *p* < .001 and 95.5% (95% CI 94.7–96.2) versus 97.2% (95% CI 96.6–97.8), *p* < 0.001, respectively. Breast PD quintile was also positively associated with cancer detected via DBT at odds ratio 1.24 (95% CI 1.09–1.42, *p* = 0.001).

**Conclusion:**

Women with the highest breast density had the greatest benefit from digital breast tomosynthesis compared with digital mammography with increased sensitivity at the cost of slightly lower specificity. These results may influence digital breast tomosynthesis’s use in an individualized screening program stratified by, for instance, breast density.

*Trial registration*. Trial registration at https://www.ClinicalTrials.gov: NCT01091545, registered March 24, 2010.

**Supplementary Information:**

The online version contains supplementary material available at 10.1186/s13058-023-01712-6.

## Introduction

Digital breast tomosynthesis (DBT) has been investigated as an alternative to digital mammography (DM) in breast cancer screening, with proven increased cancer detection rates (CDR) in several prospective trials [1]. However, the impact of DBT on recall rates has varied across different studies and screening settings [1]. Women with high breast density seem to particularly benefit from DBT due to its reduction of the overlapping tissue effect, as compared with DM [2]. Women with high breast density also have a higher risk of breast cancer, missed cancers, and false positive (FP) findings with DM compared with women with low breast density [3–5].

Radiologists commonly classify breast density into four categories according to the Breast Imaging Reporting and Data System (BI-RADS) [6]. Yet, this categorization is associated with both intra- and interobserver variations [7, 8]. In previous studies investigating DBT, breast density was often dichotomized into dense and non-dense [9]. A more detailed assessment of breast density might better capture the risk of developing breast cancer and address reduced sensitivity of cancer detection from the overlapping tissue effect [10]. Several automated quantitative breast density assessment software algorithms have been developed with the aim to primarily reduce observer variability [11]. One such software is the Laboratory for Individualized Breast Radiodensity Assessment (LIBRA) [12, 13].

Screening with DBT improves CDR compared with DM in women with dense breasts [9]. However, results from more detailed density sub-analyses in prospective trials with either BI-RADS density classification or automated software breast density assessment have shown inconsistent CDR results and recall rates in different density subgroups and most data are from American rather than European material [9, 14–16]. Accordingly, more information, especially from European data is needed. Younger women also generally have higher breast density, and DM’s sensitivity for breast cancer detection in this population is lower compared with older women [4, 17]. Further, density sub-analyses from previous prospective DBT screening trials have not included women 40–49 years old [14–16].

The prospective Malmö Breast Tomosynthesis Screening Trial compared one-view wide angle DBT alone to two-view DM and included women 40–74 years old [18]. The purpose of this current study is to evaluate which breast density subgroups, as assessed by automatic software, that have the greatest benefit from digital breast tomosynthesis compared with digital mammography in the Malmö Breast Tomosynthesis Screening Trial, with a separate evaluation for women aged 40–49 years.

## Materials and methods

### Study participants

**Fig. 1:**
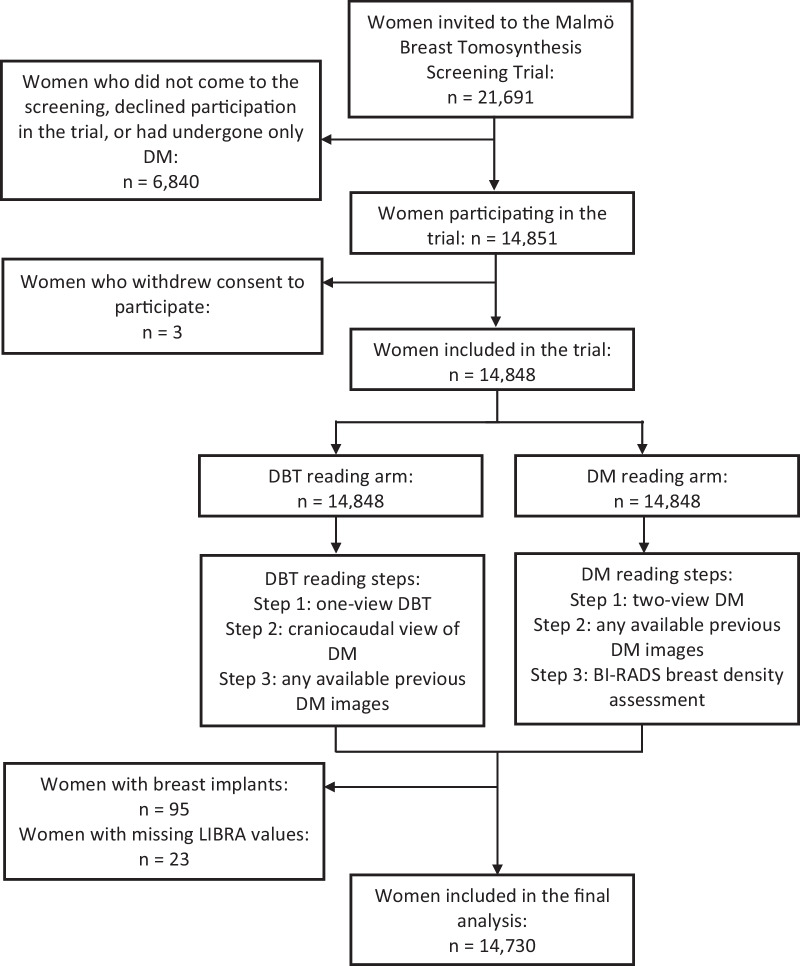
Flowchart of Malmö Breast Tomosynthesis Screening Trial participants and reading arms. *DM* digital mammography; *DBT* digital breast tomosynthesis; *BI-RADS* Breast Imaging Reporting and Data System 4^th^ ed. *LIBRA* Laboratory for Individualized Breast Radiodensity Assessment

**Fig. 2:**
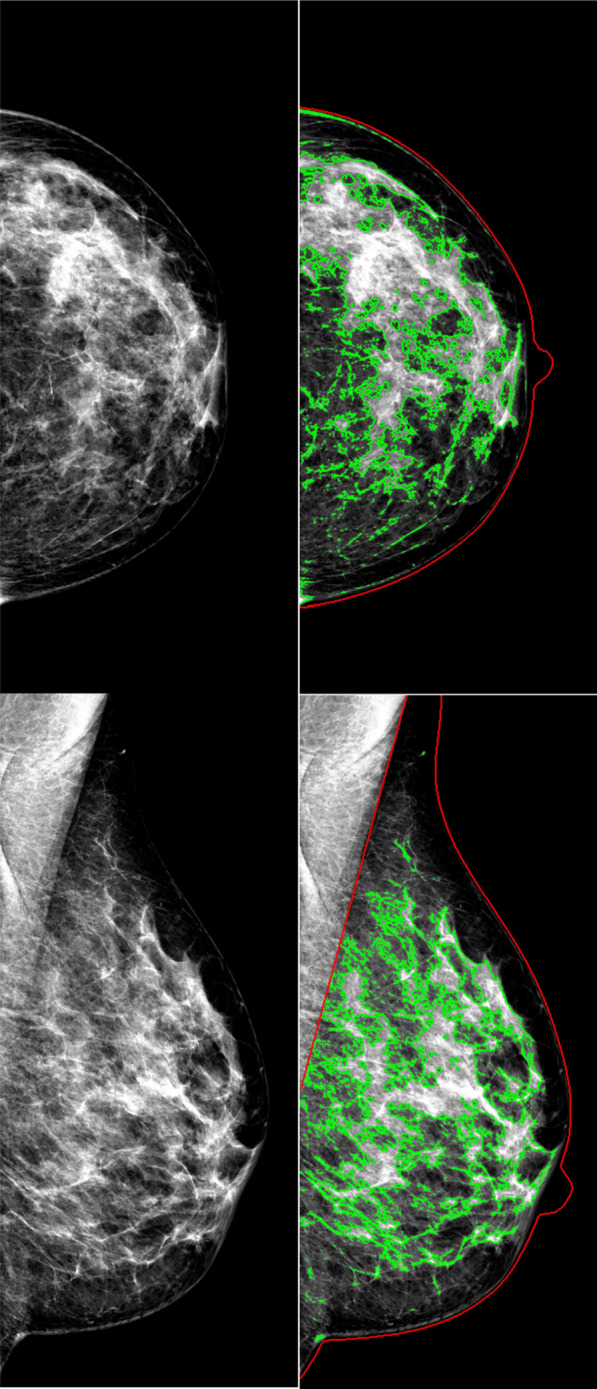
Participant images with density assessment. Images from the Laboratory for Individualized Breast Radiodensity Assessment (LIBRA) of a woman without cancer, 47 years old, who participated in the Malmö Breast Tomosynthesis Screening Trial. The woman was not recalled from screening. Breast density assessment with the LIBRA showed breast density corresponding to the fourth quintiles of both breast percent density and absolute dense area. Left images show the craniocaudal (upper) and mediolateral oblique (lower) view from digital mammography without density assessment. Right images show the same projections with density assessment. The total breast areas are marked in red and the dense areas in green

The prospective Malmö Breast Tomosynthesis Screening Trial was conducted between January 27, 2010 and February 13, 2015 at Skåne University Hospital in Malmö, Sweden. This secondary analysis was pre-specified and received ethical approval from the local ethics committee at Lund University (Dnr 2009/770; trial protocol at https://www.ClinicalTrials.gov: NCT01091545). A random sample of 21,691 women aged 40–74 years old were selected from the Malmö screening registry, asked to participate in the trial, and enrolled after providing their written informed consent (Fig. 1). Exclusion criteria were pregnancy and non-Swedish or non-English speakers. One-view (mediolateral oblique) wide angle DBT and two-view (mediolateral oblique and craniocaudal) DM images were acquired at one screening occasion with Mammomat Inspiration (Siemens Healthineers, Erlangen, Germany). The authors had full control of the data and all information submitted for publication, and none were employed by Siemens Healthineers. Seven radiologists (among them SZ) with breast imaging experience ranging from 2 to 40 years participated in the screen reading. Five of the readers had a screen reading volume of over 5000 screen examinations per year. All images were read in two separate reading arms, the DM reading arm and the DBT reading arm, with double reading in each arm and consensus meetings taking place before recall. The participants could be recalled from one or both reading arms (Fig. 1) [18, 19]. Breast density categorization within the trial was performed according to BI-RADS breast density 4th Ed categories [6] for all participating women by the first reader as part of the DM reading arm. The study sample was investigated in several previous publications (Additional file 1), though screening performance had not been investigated by automatically assessing breast density. Breast density was retrospectively assessed with the automated software LIBRA for this study (Fig. 2). Breast area and absolute dense area (DA) were analyzed for each processed DM view, resulting in four analyzed images per woman (two in women with one breast) that were combined for a mean value. The mean value of breast percent density (PD) was calculated by dividing DA by breast area. Final exclusion criteria were inability of LIBRA to perform an analysis and the presence of breast implants.

### Definitions

Previous screening was defined as a woman who had participated in the regional screening program in Skåne, Sweden in 2005 or later. Menopausal status was defined by age at DBT screening as premenopausal (< 55 years) or postmenopausal (≥ 55 years) [20].

### Study outcomes

Outcomes, calculated per woman, were sensitivity, specificity, and CDR for breast cancer per 1,000 women screened, as well as FP rate, recall rate, biopsy rate, positive predictive value for recall, and positive predictive value for biopsy. A subgroup analysis was conducted for women aged 40–49 years.

### Statistics

The study participants were divided into quintiles of PD and DA per increasing density. The outcomes of the DBT reading arm were compared with those of the DM reading arm for each breast density quintile. The density subgroups were not pre-specified in the study protocol. The sensitivity and specificity of DBT and DM were compared per each quintile and the overall study sample with McNemar’s test in SPSS Statistics for Windows (version 26, 2019, IBM Corp., Armonk, NY, USA). Logistic regression analyses were also performed in SPSS to analyze the relation between cancer detected with DBT, cancer detected with DM, FP with DBT, and FP with DM with PD or DA quintiles, adjusting for menopausal status and previous screening to generate odds ratios (OR) and 95% confidence intervals (CI). DBT and DM outcomes were calculated for each quintile using Epitools (Sergeant, ESG, 2018, Ausvet; available at: http://epitools.ausvet.com.au), presented with 95% CI. An exploratory test to analyze which quintiles had the largest difference in CDR with the use of DBT compared with DM was performed. Analyses were also performed per BI-RADS density category. Subgroup analyses for women aged 40–49 were presented descriptively for sensitivity, specificity, and CDR in new PD and DA quintiles^40−49^ and BI-RADS density categories. An alpha value of 0.05 was considered significant. A Bonferroni correction for multiple testing with six tests, 5 quintiles and overall, (five tests with BI-RADS density, 4 categories and overall) was used for McNemar’s test (alpha after correction 0.0083 and 0.01, respectively).

## Results

### Participant characteristics

This study included 14,730 women after exclusions (95 due to breast implants and 23 due to missing LIBRA values) (Fig. 1) at a median age at inclusion of 58 years (inter-quartile range = 16). Further descriptive data are presented in Table 1. One woman, later presenting with interval cancer, was recalled from the screening examination but without any cancer found at follow-up. This woman is included both as an FP and as a participant with interval cancer.

**Table 1:** Descriptive data of the study population

Parameter	Study population
Women with valid LIBRA score *n* (%)	14,730 (100%)
Recalled women with cancer or interval cancer *n* (%)	158 (1.1%)
Recalled women with cancer *n* (%)	136 (0.9%)
Women with interval cancer *n* (%)	22 (0.1%)
Recalled women *n* (%)	655 (4.4%)
Women biopsied *n* (%)	344 (2.3%)
False positives *n* (%)	519 (3.5%)
PPV-1 (Cancer % of recalled women)	136/655 (20.8%)
PPV-3 (Cancer % of women biopsied)	136/344 (39.5%)
Breast percent density (%)	
Mean (SD)	27.2% (16.5)
Median (IQR)	21.6% (21.9)
Absolute dense area (cm^2^)	
Mean (SD)	37.9 cm^2^ (18.9)
Median (IQR)	33.2 cm^2^ (22.1)
BI-RADS density categories (valid *n* = 13,801)^a^ *n* (%)	
1	2313 (16.8%)
2	5347 (38.7%)
3	4911 (35.6%)
4	1230 (8.9%)
Age (years)	
Median (IQR)	58 (16)
Premenopausal (< 55 years) *n* (%)	6360 (43.2%)
Postmenopausal (≥ 55 years) *n* (%)	8370 (56.8%)
Previous screening *n* (%)	13,231 (89.8%)
No previous screening *n* (%)	1499 (10.2%)

*LIBRA* Laboratory for Individualized Breast Radiodensity Assessment; *PPV-1* positive predictive value of recall; *PPV-3* positive predictive value of biopsy; *SD* standard deviation; *IQR* interquartile range

^a^In total, 929 women did not have a recorded Breast Imaging Reporting and Data System (BI-RADS) 4^th^ ed. Breast density measurement

### Breast percent density and absolute dense area

The median PD and DA were 21.6% and 33.2 cm^2^, respectively. Each quintile contained 2945–2947 women. Two women at the cut-off value between quintiles 3 and 4 had an equal DA. Descriptive data for all quintiles are presented in Table 2.

**Table 2:** **a** Descriptive statistics of breast percent density quintiles **b** Descriptive statistics of absolute dense area quintiles

Quintile	Women (*n*)	PD (%)	BI-RADS Density category (mean)	Age in years (mean)	No previous screening *n* (%)	Premenopausal *n* (%)
*a*						
PD 1	2946	< 13.37%	1.5	62	154/2946 (5.2%)	619/2946 (21.0%)
PD 2	2946	13.37–18.40%	1.9	60	199/2946 (6.8%)	904/2946 (30.7%)
PD 3	2946	18.40–26.12%	2.4	58	255/2946 (8.7%)	1127/2946 (38.3%)
PD 4	2946	26.12–40.98%	2.8	56	368/2946 (12.5%)	1500/2946 (50.9%)
PD 5	2946	> 40.98%	3.3	50	523/2946 (17.8%)	2210/2946 (75.0%)

Quintile	Women (*n*)	DA (cm^2^)	BI-RADS Density category (mean)	Age in years (mean)	No previous screening *n* (%)	Premenopausal *n* (%)
*b*						
DA 1	2946	< 22.86 cm^2^	1.7	62	178/2946 (6.0%)	649/2946 (22.0%)
DA 2	2946	22.86–29.25 cm^2^	2.0	60	205/2946 (7.0%)	892/2946 (30.3%)
DA 3	2947	29.25–37.67 cm^2^	2.3	58	265/2947 (9.0%)	1220/2947 (41.4%)
DA 4	2945	37.67–50.95 cm^2^	2.7	55	361/2945 (12.3%)	1574/2945 (53.5%)
DA 5	2946	> 50.95 cm^2^	3.1	52	490/2946 (16.6%)	2025/2946 (68.7%)

*PD* breast percent density; *BI-RADS* Breast Imaging Reporting and Data System 4^th^ ed; *DA* absolute dense area

### Sensitivity

**Fig. 3:**
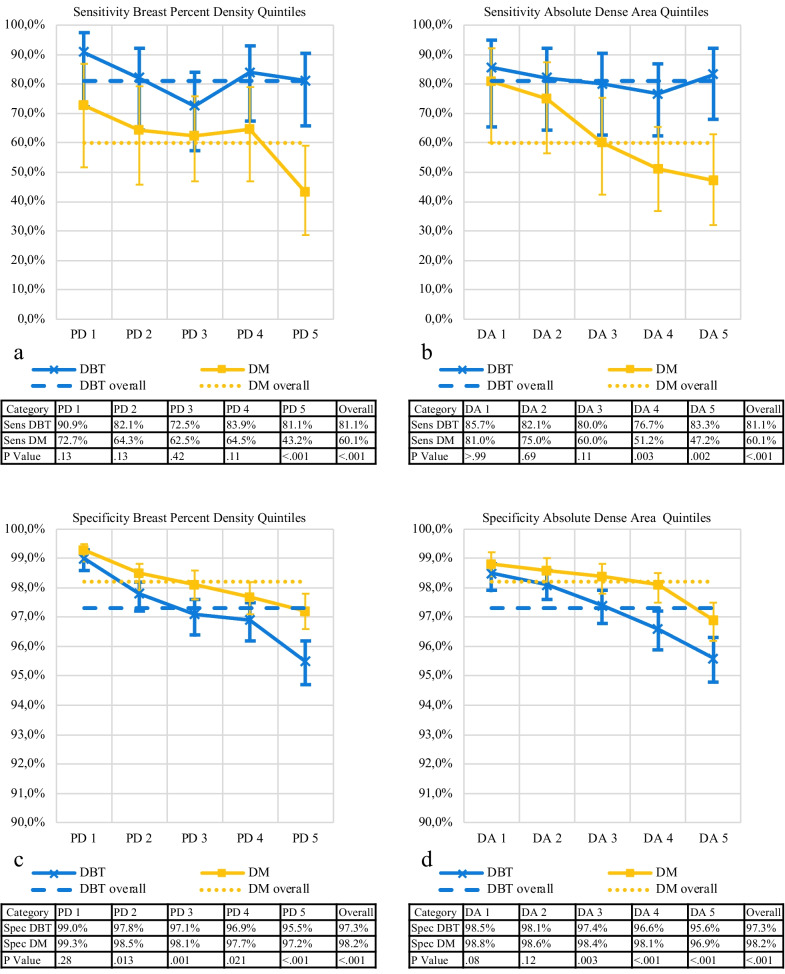
**a**–**d** Graphs of sensitivity and specificity. Graph of (a and b) sensitivity (sens) and (c and d) specificity (spec) of breast percent density (PD) and absolute dense area (DA) in all quintiles for digital breast tomosynthesis (DBT) and digital mammography (DM), with 95% confidence intervals as vertical lines. Dotted lines mark overall sensitivity and specificity for DBT and DM

Sensitivity was higher for DBT compared with DM for all PD quintiles, significantly for the highest quintile (81.1% (95% CI 65.8–90.5) vs 43.2% (95% CI 28.7–59.1), *p* < 0.001; Fig. 3 and Table 3). The DA quintiles had similar results, with significance for quintile 4 (76.7% (95% CI 62.7–86.8) vs 51.2% (95% CI 36.8–65.4), *p* = 0.003) and quintile 5 (83.3% (95% CI 68.1–92.1) vs 47.2% (95% CI 32.0–63.0), *p* = 0.002). The largest absolute difference in sensitivity between DBT and DM emerged in quintile 5 for both PD (37.9 percentage points (95% CI 15.8–60.0)) and DA (36.1 percentage points (95% CI 14.1–58.1)).

**Table 3:** Sensitivity of digital breast tomosynthesis and digital mammography in all quintiles

Quintile	Sensitivity DBT % (n) (95% CI)	Sensitivity DM % (n) (95% CI)	Difference DBT vs DM percentage points (95% CI)	*p* Value
PD 1	90.9% (20/22) (72.2–97.5)	72.7% (16/22) (51.8–86.8)	18.2 ( − 4.6–41.0)	0.13
PD 2	82.1% (23/28) (64.4–92.1)	64.3% (18/28) (45.8–79.3)	17.8 ( − 5.4–41.0)	0.13
PD 3	72.5% (29/40) (57.2–83.9)	62.5% (25/40) (47.0–75.8)	10.0 ( − 10.5–30.5)	0.42
PD 4	83.9% (26/31) (67.4–92.9)	64.5% (20/31) (46.9–78.9)	19.4 ( − 2.4–41.2)	0.11
PD 5	81.1% (30/37) (65.8–90.5)	43.2% (16/37) (28.7–59.1)	37.9 (15.8–60.0)	< 0.001
Overall	81.0% (128/158) (74.2–86.4)	60.1% (95/158) (52.3–67.4)	20.9 (10.8–30.9)	< 0.001

DA 1	85.7% (18/21) (65.4–95.0)	81.0% (17/21) (60.0–92.3)	4.8 ( − 17.8–27.3)	> 0.99
DA 2	82.1% (23/28) (64.4–92.1)	75.0% (21/28) (56.6–87.3)	7.1 ( − 14.4–28.6)	0.69
DA 3	80.0% (24/30) (62.7–90.5)	60.0% (18/30) (42.3–75.4)	20.0 ( − 3.2–43.2)	0.11
DA 4	76.7% (33/43) (62.3–86.8)	51.2% (22/43) (36.8–65.4)	25.6 (5.3–45.9)	0.003
DA 5	83.3% (30/36) (68.1–92.1)	47.2% (17/36) (32.0–63.0)	36.1 (14.1–58.1)	0.002
Overall	81.0% (128/158) (74.2–86.4)	60.1% (95/158) (52.3–67.4)	20.9 (10.8–30.9)	< 0.001

*DBT* digital breast tomosynthesis; *CI* confidence interval; *DM* digital mammography; *PD* breast percent density; *DA* absolute dense area

### Specificity

Specificity was lower for DBT compared with DM for all PD quintiles, significant for quintile 3 (97.1% (95% CI 96.4–97.6) vs 98.1% (95% CI 97.6–98.1), *p* = 0.001) and quintile 5 (95.5% (95% CI 94.7–96.2) vs 97.2% (95% CI 96.6–97.8), *p* < 0.001; Additional file 2: Table S1). The DA quintiles revealed similar results, with significantly lower specificity for DBT compared with DM for quintile 3 (97.4% (95% CI 96.8–97.9) vs 98.4% (95% CI 97.8–98.8), *p* = 0.003), quintile 4 (96.6% (95% CI 95.9–97.2) vs 98.1% (95% CI 97.5–98.5), *p* < 0.001), and quintile 5 (95.6% (95% CI 94.8–96.3) vs 96.9% (95% CI 96.2–97.5), *p* < 0.001). The largest absolute difference in specificity between DBT and DM was seen in PD quintile 5 (1.7 percentage points (95% CI 0.8–2.7)) and DA quintile 4 (1.4 percentage points (95% CI 0.6–2.3)).

### Logistic regression

In the logistic regression models, after adjustment for menopausal status and previous screening, higher PD and DA quintiles were associated with cancer detected with DBT (OR 1.24 (95% CI 1.09–1.42, *p* = 0.001) and OR 1.28 (95% CI 1.12–1.46, *p* < 0.001), respectively). This relationship was not seen for cancer detected with DM for neither PD nor DA (Table 4). Higher PD and DA quintiles were also associated with FP for both DBT (OR 1.27 (95% CI 1.17–1.38, *p* < 0.001) and OR 1.23 (95% CI 1.14–1.33, *p* < 0.001), respectively) and DM (OR 1.24 (95% CI 1.13–1.37, *p* < 0.001) and OR 1.20 (95% CI 1.10–1.32, *p* < 0.001), respectively). In the logistic regression, previous screening did not significantly affect cancer detection or FP after adjustments. Postmenopausal women had a higher OR for cancer detection and a lower OR for FP with both DBT and DM after adjusting for previous screening and density by PD or DA.

**Table 4:** Multivariable logistic regression for detected breast cancers and false positive recall

Characteristics	Cancer DBT		Cancer DM		FP DBT		FP DM	
	Adjusted OR (95% CI)	*p* Value	Adjusted OR (95% CI)	*p* Value	Adjusted OR (95% CI)	*p* Value	Adjusted OR (95% CI)	*p* Value
PD 1	1—Reference		1—Reference		1—Reference		1—Reference	
PD 2	1.25 (0.68–2.27)	0.48	1.21 (0.62–2.39)	0.58	2.23 (1.43–3.48)	< 0.001	2.06 (1.22–3.47)	0.007
PD 3	1.68 (0.95–2.98)	0.08	1.80 (0.96–3.39)	0.07	1.85 (1.85–4.39)	< 0.001	2.39 (1.43–3.97)	0.001
PD 4	1.69 (0.94–3.05)	0.08	1.62 (0.83–3.15)	0.16	2.82 (1.83–4.35)	< 0.001	2.84 (1.72–4.68)	<0 .001
PD 5	2.56 (1.41–4.65)	0.002	1.69 (0.82–3.50)	0.16	3.67 (2.39–5.63)	<0 .001	3.06 (1.85–5.05)	< 0.001
Trend PD	1.24 (1.09–1.42)	0.001	1.15 (0.98–1.34)	0.08	1.27 (1.17–1.38)	< 0.001	1.24 (1.13–1.37)	< 0.001
Postmenopausal	3.04 (1.94–4.78)	< 0.001	2.71 (1.60–4.56)	< 0.001	0.63 (0.50–0.78)	< 0.001	0.66 (0.50–0.86)	0.002
Previous Screening	0.81 (0.42–1.56)	0.52	1.34 (0.53–3.42)	.54	0.82 (0.62–1.09)	0.17	0.82 (0.58–1.16)	0.27

DA 1	1—Reference		1—Reference		1—Reference		1—Reference	
DA 2	1.37 (0.74–2.54)	0.32	1.32 (0.69–2.50)	0.40	1.17 (0.78–1.74)	0.45	1.16 (0.73–1.83)	0.53
DA 3	1.47 (0.85–2.90)	0.15	1.24 (0.64–2.41)	0.53	1.50 (1.03–2.19)	0.03	1.28 (0.82–2.00)	0.28
DA 4	2.43 (1.36–4.35)	0.003	1.70 (0.89–3.23)	0.11	1.85 (1.29–2.66)	0.001	1.41 (0.91–2.18)	0.13
DA 5	2.60 (1.42–4.76)	0.002	1.54 (0.77–3.10)	0.22	2.25 (1.57–3.22)	< 0.001	2.13 (1.40–3.23)	< 0.001
Trend DA	1.28 (1.12–1.46)	< 0.001	1.12 (0.96–1.30)	0.14	1.23 (1.14–1.33)	< 0.001	1.20 (1.10–1.32)	< 0.001
Postmenopausal	3.00 (1.93–4.67)	< 0.001	2.67 (1.60–4.47)	< 0.001	0.60 (0.48–0.75)	< 0.001	0.63 (0.48–0.83)	0.001
Previous Screening	0.80 (0.42–1.55)	0.51	1.34 (0.52–3.41)	0.54	0.81 (0.61–1.08)	0.15	0.82 (0.58–1.16)	0.26

*DBT* digital breast tomosynthesis; *OR* odds ratio; *CI* confidence interval; *DM* digital mammography; *FP* false positive; *PD* breast percent density; *DA* absolute dense area

### Cancer detection rate and false positives

**Fig. 4:**
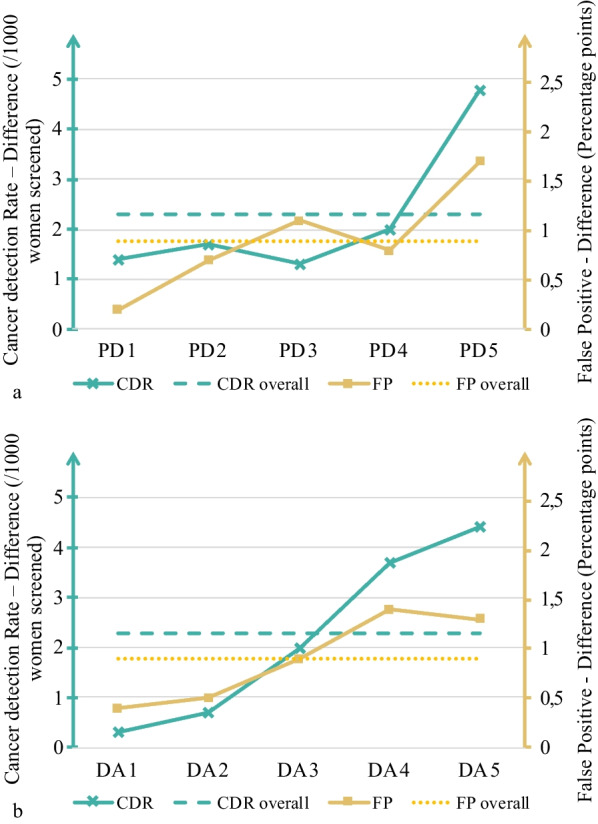
**a** and **b** Graphs of differences in cancer detection and false positives. Graph of differences in cancer detection rate (CDR) per 1000 women screened and false positives (FP) in percentage points between digital breast tomosynthesis and digital mammography for all (**a**) breast percent density (PD) and (**b**) absolute dense area (DA) quintiles. Dotted lines mark overall difference in CDR and FP

CDR was higher with DBT compared with DM in all five quintiles, both for PD and DA. However, the CI for difference included zero for all quintiles except the highest PD quintile (Fig. 4 and Additional file 2: Table S2). The largest difference between DBT and DM was found in the highest PD and DA quintiles, with 4.8 (95% CI 0.3–9.3) and 4.4 (95% CI  − 0.1–9.0) additional cancer detections per 1,000 women screened, respectively. FP rates were also higher for DBT compared with DM for all PD and DA quintiles, although with CI for difference overlapping zero for PD quintiles 1 and 4 and DA quintiles 1 and 2.

### Recall, biopsy rate, positive predictive value for recall, and positive predictive value for biopsy

**Fig. 5:**
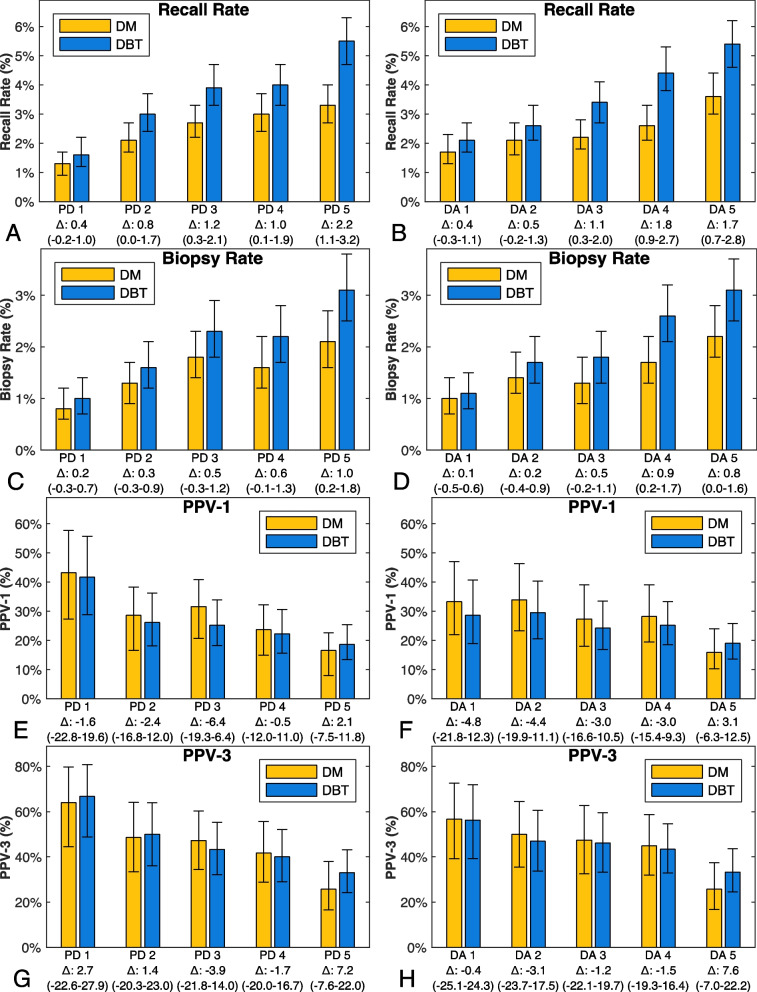
Bar charts of recall rate, biopsy rate and positive predictive values. Bar charts of (**A** and **B**) recall rate, (**C** and **D**) biopsy rate, (**E** and **F**) positive predictive value of recall (PPV-1), and (**G** and **H**) positive predictive value of biopsy (PPV-3) of breast percent density (PD) and absolute dense area (DA) in all quintiles for digital breast tomosynthesis (DBT) and digital mammography (DM), with 95% confidence intervals (CI) in vertical lines. The difference (Δ) between DBT and DM are presented in percentage points with 95% CI in parenthesis

Recall rates were highest for both DBT and DM in the highest PD and DA quintiles (Fig. 5). Biopsy rates were higher for DBT compared with DM for all PD and DA quintiles, albeit with CI for difference overlapping zero for PD quintiles 1–4 and DA quintiles 1–3 (Additional file 2: Table S3). The positive predictive values for recall and biopsy were similar between DBT and DM across all PD and DA quintiles.

### Exploratory analysis

An exploratory test analyzed which quintiles had the largest difference in CDR when using DBT. For PD, the largest gain was in quintile 5 alone, so no further testing was done. For DA, the largest gain in CDR occurred in quintiles 4 and 5. When these quintiles were analyzed together, the incremental CDR was 4.1 (95% CI 0.7–7.4) additional cancer detections per 1,000 women screened for DBT compared with DM. The corresponding incremental FP rate for the DA quintiles with DBT compared with DM was 1.4 percentage points (95% CI 0.7–2.0).

### Women 40–49 years old

For women aged 40–49 years, the median PD and DA were 35.8% and 43.9 cm^2^, respectively. Additional file 2: Table S4 provides the descriptive data of the PD and DA quintiles^40−49^ among this study subgroup. Sensitivity, specificity, and CDR in all PD and DA quintiles^40−49^ as well as in BI-RADS density categories for DBT and DM are available in Table 5 and Additional file 2: Table S5. Overall sensitivity was higher (82.1% (95%CI 64.4–92.1) vs 53.6% (95% CI 35.8–70.5), *p* = 0.02) and overall specificity lower (95.8% (95% CI 95.2–96.4) vs 97.0% (95% CI 96.4–97.4); *p* < 0.001) for DBT compared with DM for women aged 40–49. Higher sensitivity and CDR but somewhat lower specificity for DBT compared with DM, as in the full study sample, occurred across most quintiles^40−49^.

**Table 5:** Sensitivity, specificity, and cancer detection rate among women 40–49 years old

Quintile^40−49^	Sensitivity DBT % (*n*) (95% CI)	Sensitivity DM % (*n*) (95% CI)	Specificity DBT % (*n*) (95% CI)	Specificity DM % (*n*) (95% CI)	CDR DBT (*n*) (95% CI)	CDR DM (*n*) (95% CI)
PD 1^40–49^	87.5% (7/8) (52.9–97.8)	75.0% (6/8) (40.9–92.9)	97.3% (795/818) (96.0–98.2)	98.3% (804/818) (97.1–99.0)	8.5 (7/826) (4.1–17.4)	7.3 (6/826) (3.3–15.8)
PD 2^40–49^	75.0% (3/4) (30.1–95.4)	50.0% (2/4) (15.0–85.0)	96.5% (793/822) (95.0–97.5)	96.8% (796/822) (95.4–97.8)	3.6 (3/826) (1.2–10.6)	2.4 (2/826) (0.7–8.8)
PD 3^40–49^	100.0% (3/3) (43.9–100.0)	66.7% (2/3) (20.8–93.9)	95.1% (783/823) (93.4–96.4)	96.5% (794/823) (95.0–97.5)	3.6 (3/826) (1.2–10.6)	2.4 (2/826) (0.7–8.8)
PD 4^40–49^	75.0% (6/8) (40.9–92.9)	25.0% (2/8) (7.2–59.1)	95.1% (778/818) (93.4–96.4)	96.3% (788/818) (94.8–97.4)	7.3 (6/826) (3.3–15.8)	2.4 (2/826) (0.7–8.8)
PD 5^40–49^	80.0% (4/5) (37.6–96.4)	60.0% (3/5) (23.1–88.2)	95.1% (781/821) (93.4–96.4)	96.8% (795/821) (95.4–97.8)	4.8 (4/826) (1.9–12.4)	3.6 (3/826) (1.2–10.6)
Overall^40−49^	82.1% (23/28) (64.4–92.1)	53.6% (15/28) (35.8–70.5)	95.8% (3931/4102) (95.2–96.4)	97.0% (3977/4102) (96.4–97.4)	5.6 (23/4130) (3.7–8.3)	3.6 (15/4130) (2.2–6.0)

DA 1^40–49^	50.0% (1/2) (9.5–90.6)	100.0% (2/2) (34.2–100.0)	97.0% (799/824) (95.6–97.9)	97.5% (803/824) (96.1–98.3)	1.2 (1/826) (0.2–6.8)	2.4 (2/826) (0.7–8.8)
DA 2^40–49^	100.0% (6/6) (61.0–100.0)	66.7% (4/6) (30.0–90.3)	96.5% (791/820) (95.0–97.5)	98.2% (805/820) (97.0–98.9)	7.3 (6/826) (3.3–15.8)	4.8 (4/826) (1.9–12.4)
DA 3^40–49^	62.5% (5/8) (30.6–86.3)	37.5% (3/8) (13.7–69.4)	96.1% (786/818) (94.5–97.2)	96.9% (793/818) (95.5–97.9)	6.1 (5/826) (2.6–14.1)	3.6 (3/826) (1.2–10.6)
DA 4^40–49^	80.0% (4/5) (37.55–96.4)	40.0% (2/5) (11.8–76.9)	95.4% (783/821) (93.7–96.6)	96.0% (788/821) (94.4–97.1)	4.8 (4/826) (1.9–12.4)	2.4 (2/826) (0.7–8.8)
DA 5^40–49^	100.0% (7/7) (64.8–100.0)	57.1% (4/7) (25.1–84.2)	94.3% (722/819) (92.5–95.7)	96.2% (788/819) (94.7–97.3)	8.5 (7/826) (4.1–17.4)	4.8 (4/826) (1.9–12.4)
Overall^40−49^	82.1% (23/28) (64.4–92.1)	53.6% (15/28) (35.8–70.5)	95.8% (3931/4102) (95.2–96.4)	97.0% (3977/4102) (96.4–97.4)	5.6 (23/4130) (3.7–8.3)	3.6 (15/4130) (2.2–6.0)

*DBT* digital breast tomosynthesis; *CI* confidence interval; *DM* digital mammography; *CDR* cancer detection rate per 1000 women screened; *PD* breast percent density; *DA* absolute dense area

### Outcome by BI-RADS density category

For completeness and reference, data outcomes by BI-RADS density category are presented in Additional file 2: Tables S6–S9. These data, featuring FP, CDR, and BI-RADS density distribution results, were published in part in previous studies [18, 21–23].

## Discussion

The diagnostic accuracy of digital breast tomosynthesis (DBT) compared with digital mammography (DM) in breast cancer screening may vary per breast density subgroup. This study thus evaluated the diagnostic accuracy of DBT and DM in the Malmö Breast Tomosynthesis Screening Trial by breast density subgroup with the automatic software the Laboratory for Individualized Breast Radiodensity Assessment (LIBRA). The largest difference in cancer detection rate (CDR) in screening with DBT and DM was found among women in the highest breast density quintile. For the 20% of women with the highest breast percent density (PD), sensitivity went from 43.2% with DM to 81.1% with DBT (*p* < 0.001), corresponding to 4.8 (95% CI 0.3–9.3) additional women with breast cancer identified per 1000 screened. The largest difference in specificity between DM and DBT, with lower results for the latter, was also seen in women in the highest PD quintile; however, specificity was still high (95.5%) for DBT. Among women aged 40–49, the sensitivity of DBT was higher compared with DM in most density categories for both PD and absolute dense area (DA).

In the USA, DBT is widely implemented in screening since several years, especially among women with dense breast. However, in 2021, the European Commission Initiative on Breast Cancer published a conditional recommendation for DBT in screening women with dense breasts, albeit with “*very low certainty of the evidence*” [24]. Both European [24] and American recommendations [25] dichotomized breast density categories. Two studies with more detailed density sub-analyses with automatic breast density assessment that analyzed data from prospective trials, the Oslo Tomosynthesis Screening Trial [14] and Tomosynthesis trial in Bergen [15], did not find a significantly higher CDR for DBT compared with DM for women with the densest breasts. However, in the Oslo Tomosynthesis Screening Trial, the higher CDR for the densest group with DBT compared to DM was of similar magnitude (21.7% (95% CI 3.0–41.9), *p* = 0.06) as the incremental rate for the subgroup with the second highest breast density (22.6% (95% CI 12.9–32.9), *p* < 0.001) [14]. In the Tomosynthesis trial in Bergen, no difference in CDR between any density subgroups in DBT and DM was found [15]. These differences in findings in comparison with this study could have derived from the smaller sample sizes of the densest subgroups in both the Oslo Tomosynthesis Screening Trial and Tomosynthesis trial in Bergen. As well as that the Tomosynthesis trial in Bergen did not find any difference in CDR overall [26], in contrast to several other European trials [1]. A detailed density sub-analysis of the prospective Tomosynthesis plus Synthesized Mammography trial, which used the BI-RADS density categorization, found a significantly higher CDR with DBT compared with DM for women with the highest breast density (OR 3.8 (95% CI 1.5–11.1)), which is in agreement with the present study’s findings [16]. Neither the Tomosynthesis trial in Bergen nor the Oslo Tomosynthesis Screening Trial found any significant difference in FP between DBT and DM among women with the highest breast density [14, 15]. These different results compared with this study could again be due to the smaller sample size among the densest subgroups and the Oslo Tomosynthesis Screening Trial’s FP rate being derived before the consensus meeting.

Automated breast density assessment enables reproducibility. LIBRA can assess breast density in both raw and processed images [12], which is beneficial since in clinical settings, it is common that only processed images are stored [27]. Whether PD or DA should be used for breast density assessment is still debatable [28], although it has been suggested that PD has a higher correlation with breast cancer risk [29]. The current study’s results showed similarities between PD and DA, but in exploratory analyses, a larger group that benefits more from DBT in terms of increased CDR could be identified with DA. Still, this study was not designed to compare the two different breast density assessment methods.

The current study does have limitations. The subgroup division and post hoc analysis were not powered in the original trial, though significant differences were still found in the higher breast density subgroups. DM’s FP rate in this trial could also be underestimated due to the DBT images available at the consensus meeting, which caused DM to be favored. The LIBRA assessments were not manually reviewed, though LIBRA has previously been validated for Siemens images, with a strong association with radiologists’ density assessments (*r* = 0.89) [20]. Images with failed LIBRA readings, due to bad positioning of the breast, were excluded in the study. However, the number of failed readings were low (*n* = 23). Further, the density measurement with LIBRA was assessed area-based from DM-images. A stronger association with breast cancer, has however, previously been shown for volumetric measurements from DBT [30]. Finally, the subgroup of women aged 40–49 was small, so these results should be interpreted with caution.

The findings in this study add important knowledge to the scarce evidence regarding DBT screening in women with the densest breasts, showing greatest impact for women in the highest breast density subgroup. To evaluate the full value of DBT in the screening program, future evaluation should assess breast density beyond binary categorization.

## Conclusion

In conclusion, women with high mammographic density, as assessed with automatic density software, had the greatest benefit from digital breast tomosynthesis screening compared with digital mammography, as it improved cancer detection for 20–40% of the screening population at the cost of a small decrease in specificity. These results may influence digital breast tomosynthesis’s use in a future individualized screening program stratified by, for instance, breast density.

### Supplementary Information

**Additional file 1**: Previously published data of study participants with explanations.

**Additional file 2**: **Table S1**. Specificity of Digital Breast Tomosynthesis and Digital Mammography in All Quintiles. **Table S2**. Cancer Detection Rate, False Positives, and Recall Rate for Digital Breast Tomosynthesis and Digital Mammography in All Quintiles. **Table S3**. Biopsy Rate, Positive Predictive Value for Recall, and Positive Predictive Value for Biopsy for Digital Breast Tomosynthesis and Digital Mammography in All Quintiles. **Table S4a**. Descriptive Statistics of Breast Percent Density Quintiles40-49 for Women 40–49 Years Old. **Table S4b**. Descriptive Statistics of Absolute Dense Area Quintiles40-49 for Women 40–49 Years Old. **Table S5**. Sensitivity, Specificity, and Cancer Detection Rate among Women 40–49 Years Old in All BI-RADS Density Categories. **Table S6.** Sensitivity and Specificity of Digital Breast Tomosynthesis and Digital Mammography in All BI-RADS Density Categories. **Table S7**. Cancer Detection Rate and False Positives for Digital Breast Tomosynthesis and Digital Mammography in All BI-RADS Density Categories. **Table S8**. Biopsy and Recall Rates for Digital Breast Tomosynthesis and Digital Mammography in All BI-RADS Density Categories. **Table S9**. Positive Predictive Values of Recall and Biopsy in Digital Breast Tomosynthesis and Digital Mammography in All BI-RADS Density Categories.

## Data Availability

The datasets generated and/or analyzed during the current study are not publicly available due containing sensitive personal data but are available from the corresponding author on reasonable request.

## Abbreviations

DBT: Digital breast tomosynthesis
DM: Digital mammography
CDR: Cancer detection rate
FP: False positive
BI-RADS: Breast imaging reporting and data system
LIBRA: Laboratory for individualized breast radiodensity assessment
DA: Absolute dense area
PD: Breast percent density
OR: Odds ratio
CI: Confidence interval
