# Supplementary material for: Impact of breast density on diagnostic accuracy in digital breast tomosynthesis versus digital mammography: results from a European screening trial
Source: Breast Cancer Res. 2023 Oct 4;25:116. doi: 10.1186/s13058-023-01712-6 (PMC10548633; doi:10.1186/s13058-023-01712-6)
Supplement: Supplementary file 1 — Additional file 1: Previously published data of study participants with explanations. [file 13058_2023_1712_MOESM1_ESM.docx]

**Previously published data of study participants**

Results from the Malmö Breast Tomosynthesis Screening Trial have previously been published regarding screening accuracy (1-3), false positive recalls (4, 5, 6), interval cancers (7, 8), tumor characteristics, radiographic appearance (9), and performance of artificial intelligence (10, 11). None of the previous publications have investigated screening performance by automatically assessed breast density. Breast Imaging Reporting and Data System 4th ed breast density categorization has previously been included; as stratification for comparison between digital breast tomosynthesis and digital mammography of false positive recalls in interim analysis (5), descriptively with numbers of cancers found with digital breast tomosynthesis and digital mammography for each density category for the full study sample (1), as stratification of performance with artificial intelligence (11), for comparison of breast density measurements with breast density assessed by automated software (12-14) and as controls for breast density change over time (15). Data for women <50 has been published for recall rate and cancer detection rate between digital breast tomosynthesis and digital mammography, without simultaneous density stratification (3).

1. Zackrisson S, Lång K, Rosso A, Johnson K, Dustler M, Fornvik D, et al. One-view breast tomosynthesis versus two-view mammography in the Malmo Breast Tomosynthesis Screening Trial (MBTST): a prospective, population-based, diagnostic accuracy study. Lancet Oncol. 2018;19(11):1493-1503 **doi**: 10.1016/s1470-2045(18)30521-7.

2. Lång K, Andersson I, Rosso A, Tingberg A, Timberg P, Zackrisson S. Performance of one-view breast tomosynthesis as a stand-alone breast cancer screening modality: results from the Malmo Breast Tomosynthesis Screening Trial, a population-based study. Eur radiol. 2016;26(1):184-190 **doi**: 10.1007/s00330-015-3803-3.

3. Libesman S, Zackrisson S, Hofvind S, Seidler AL, Bernardi D, Lång K, et al. An individual participant data meta-analysis of breast cancer detection and recall rates for digital breast tomosynthesis versus digital mammography population screening. Clin Breast Cancer. 2022;22(5):e647-e654 **doi**: 10.1016/j.clbc.2022.02.005.

4. Lång K, Nergården M, Andersson I, Rosso A, Zackrisson S. False positives in breast cancer screening with one-view breast tomosynthesis: An analysis of findings leading to recall, work-up and biopsy rates in the Malmö Breast Tomosynthesis Screening Trial. Eur radiol. 2016;26(11):3899-3907 **doi**: 10.1007/s00330-016-4265-y.

5. Rosso A, Lång K, Petersson IF, Zackrisson S. Factors affecting recall rate and false positive fraction in breast cancer screening with breast tomosynthesis – A statistical approach. The Breast. 2015;24(5):680-6 **doi**: 10.1016/j.breast.2015.08.007.

6. Johnson K, Olinder J, Rosso A, Andersson I, Lång K, Zackrisson S. False-positive recalls in the prospective Malmö Breast Tomosynthesis Screening Trial. Eur radiol. 2023 **doi**: 10.1007/s00330-023-09705-x.

7. Johnson K, Lång K, Ikeda DM, Åkesson A, Andersson I, Zackrisson S. Interval Breast Cancer Rates and Tumor Characteristics in the Prospective Population-based Malmö Breast Tomosynthesis Screening Trial. Radiology. 2021;299(3):559-67 **doi**: 10.1148/radiol.2021204106.

8. Houssami N, Hofvind S, Soerensen AL, Robledo KP, Hunter K, Bernardi D, et al. Interval breast cancer rates for digital breast tomosynthesis versus digital mammography population screening: An individual participant data meta-analysis. EClinicalMedicine. 2021;34:100804 **doi**: 10.1016/j.eclinm.2021.100804.

9. Johnson K, Zackrisson S, Rosso A, Sartor H, Saal LH, Andersson I, et al. Tumor Characteristics and Molecular Subtypes in Breast Cancer Screening with Digital Breast Tomosynthesis: The Malmö Breast Tomosynthesis Screening Trial. Radiology. 2019;293(2):273-81 **doi**: 10.1148/radiol.2019190132.

10. Lång K, Dustler M, Dahlblom V, Åkesson A, Andersson I, Zackrisson S. Identifying normal mammograms in a large screening population using artificial intelligence. Eur radiol. 2021;31(3):1687-92 **doi**: 10.1007/s00330-020-07165-1.

11. Dahlblom V, Andersson I, Lång K, Tingberg A, Zackrisson S, Dustler M. Artificial Intelligence Detection of Missed Cancers at Digital Mammography That Were Detected at Digital Breast Tomosynthesis. Radiol Artif Intell. 2021;3(6):e200299 **doi**: 10.1148/ryai.2021200299.

12. Sartor H, Lång K, Rosso A, Borgquist S, Zackrisson S, Timberg P. Measuring mammographic density: comparing a fully automated volumetric assessment versus European radiologists’ qualitative classification. Eur radiol. 2016;26(12):4354-60 **doi**: 10.1007/s00330-016-4309-3.

13. Förnvik D, Förnvik H, Fieselmann A, Lång K, Sartor H. Comparison between software volumetric breast density estimates in breast tomosynthesis and digital mammography images in a large public screening cohort. Eur radiol. 2019;29(1):330-6 **doi**: 10.1007/s00330-018-5582-0.

14. Fieselmann A, Förnvik D, Förnvik H, Lång K, Sartor H, Zackrisson S, et al. Volumetric breast density measurement for personalized screening: accuracy, reproducibility, consistency, and agreement with visual assessment. J Med Imaging (Bellingham). 2019;6(3):031406 **doi**: 10.1117/1.Jmi.6.3.031406.

15. Sartor H, Kontos D, Ullén S, Förnvik H, Förnvik D. Changes in breast density over serial mammograms: A case-control study. Eur J Radiol. 2020; 127:108980 **doi**: 10.1016/j.ejrad.2020.108980.
