# Supplementary material for: Impact of breast density on diagnostic accuracy in digital breast tomosynthesis versus digital mammography: results from a European screening trial
Source: Breast Cancer Res. 2023 Oct 4;25:116. doi: 10.1186/s13058-023-01712-6 (PMC10548633; doi:10.1186/s13058-023-01712-6)
Supplement: Supplementary file 2 — Additional file 2: Table S1. Specificity of Digital Breast Tomosynthesis and Digital Mammography in All Quintiles. Table S2. Cancer Detection Rate, False Positives, and Recall Rate for Digital Breast Tomosynthesis and Digital Mammography in All Quintiles. Table S3. Biopsy Rate, Positive Predictive Value for Recall, and Positive Predictive Value for Biopsy for Digital Breast Tomosynthesis and Digital Mammography in All Quintiles. Table S4a. Descriptive Statistics of Breast Percent Density Quintiles40-49 for Women 40–49 Years Old. Table S4b. Descriptive Statistics of Absolute Dense Area Quintiles40-49 for Women 40–49 Years Old. Table S5. Sensitivity, Specificity, and Cancer Detection Rate among Women 40–49 Years Old in All BI-RADS Density Categories. Table S6. Sensitivity and Specificity of Digital Breast Tomosynthesis and Digital Mammography in All BI-RADS Density Categories. Table S7. Cancer Detection Rate and False Positives for Digital Breast Tomosynthesis and Digital Mammography in All BI-RADS Density Categories. Table S8. Biopsy and Recall Rates for Digital Breast Tomosynthesis and Digital Mammography in All BI-RADS Density Categories. Table S9. Positive Predictive Values of Recall and Biopsy in Digital Breast Tomosynthesis and Digital Mammography in All BI-RADS Density Categories. [file 13058_2023_1712_MOESM2_ESM.docx]

**Supplemental Table 1 Specificity of Digital Breast Tomosynthesis and Digital Mammography in All Quintiles**

| **Quintile** | **Specificity DBT**  **% (n) (95% CI)** | **Specificity DM**  **% (n) (95% CI)** | **Difference DBT vs DM percentage points (95% CI)** | **P Value** |
| --- | --- | --- | --- | --- |
| **PD 1** | 99.0% (2896/2924)  (98.6-99.3) | 99.3% (2903/2924)  (98.9-99.5) | -0.2  (-0.7-0.2) | .28 |
| **PD 2** | 97.8% (2853/2918)  (97.2-98.2) | 98.5% (2873/2918)  (97.9-98.8) | -0.7  (-1.4-0.0) | .01 |
| **PD 3** | 97.1% (2821/2906)  (96.4-97.6) | 98.1% (2852/2906)  (97.6-98.6) | -1.1  (-1.9-(-0.3)) | .001 |
| **PD 4** | 96.9% (2824/2915)  (96.2-97.5) | 97.7% (2847/2915)  (97.1-98.2) | -0.8  (-1.6-0.1) | .02 |
| **PD 5** | 95.5% (2778/2909)  (94.7-96.2) | 97.2% (2828/2909)  (96.6-97.8) | -1.7  (-2.7-(-0.8)) | < .001 |
| **Overall** | 97.3% (14172/14572)  (97.0-97.5) | 98.2% (14303/14572)  (97.9-98.4) | -0.9  (-1.2-(-0.6)) | < .001 |
|  |  |  |  |  |
| **DA 1** | 98.5% (2880/2925)  (97.9- 98.8) | 98.8% (2891/2925)  (98.4-99.2) | -0.4  (-1.0-0.2) | .08 |
| **DA 2** | 98.1% (2864/2918)  (97.6-98.6) | 98.6% (2877/2918)  (98.1-99.0) | -0.4  (-1.1-0.2) | .12 |
| **DA 3** | 97.4% (2842/2917)  (96.8-97.9) | 98.4% (2869/2917)  (97.8-98.8) | -1.0  (-1.7-(-0.2)) | .003 |
| **DA 4** | 96.6% (2804/2902)  (95.9-97.2) | 98.1% (2846/2902)  (97.5-98.5) | -1.4  (-2.3-(-0.6)) | < .001 |
| **DA 5** | 95.6% (2782/2910)  (94.8-96.3) | 96.9% (2820/2910)  (96.2-97.5) | -1.3  (-2.3-(-0.3)) | < .001 |
| **Overall** | 97.3% (14172/14572)  (97.0-97.5) | 98.2% (14303/14572)  (97.9-98.4) | -0.9  (-1.2-(-0.6)) | < .001 |

DBT = digital breast tomosynthesis. CI = confidence interval. DM = digital mammography. PD = breast percent density. DA = absolute dense area.

**Supplemental Table 2 Cancer Detection Rate, False Positives, and Recall Rate for Digital Breast Tomosynthesis and Digital Mammography in All Quintiles**

| **Quintile** | **CDR DBT**  **(n) (95% CI)** | **CDR DM**  **(n) (95% CI)** | **Difference CDR**  **(95% CI)** | **FP DBT**  **% (n) (95% CI)** | **FP DM**  **% (n) (95% CI)** | **Difference FP (pp)**  **(95% CI)** | **Recall DBT**  **% (n) (95% CI)** | **Recall DM**  **% (n) (95% CI)** | **Difference Recall (pp)**  **(95% CI)** |
| --- | --- | --- | --- | --- | --- | --- | --- | --- | --- |
| **PD 1** | 6.8 (20/2946)  (4.4-10.5) | 5.4 (16/2946)  (3.3-8.8) | 1.4  (-2.6-5.4) | 1.0% (28/2946)  (0.7-1.4) | 0.7% (21/2946)  (0.5-1.1) | 0.2  (-0.2-0.7) | 1.6% (48/2946)  (1.2-2.2) | 1.3% (37/2946)  (0.9-1.7) | 0.4  (-0.2-1.0) |
| **PD 2** | 7.8 (23/2946)  (5.2-11.7) | 6.1 (18/2946)  (3.9-9.6) | 1.7  (-2.5-5.9) | 2.2% (65/2946)  (1.7-2.8) | 1.5% (45/2946)  (1.1-2.0) | 0.7  (0.0-1.4) | 3.0% (88/2946)  (2.4-3.7) | 2.1% (63/2946)  (1.7-2.7) | 0.8  (0.0-1.7) |
| **PD 3** | 9.8 (29/2946)  (6.9-14.1) | 8.5 (25/2946)  (5.8-12.5) | 1.3  (-3.6-6.2) | 2.9% (86/2946)  (2.4-3.6) | 1.8% (54/2946)  (1.4-2.4) | 1.1  (0.3-1.9) | 3.9% (115/2946)  (3.3-4.7) | 2.7% (79/2946)  (2.2-3.3) | 1.2  (0.3-2.1) |
| **PD 4** | 8.8 (26/2946)  (6.0-12.9) | 6.8 (20/2946)  (4.4-10.5) | 2.0  (-2.5-6.5) | 3.1% (91/2946)  (2.5-3.8) | 2.3% (68/2946)  (1.8-2.9) | 0.8  (-0.1-1.6) | 4.0% (117/2946)  (3.3-4.7) | 3.0% (88/2946)  (2.4-3.7) | 1.0  (0.1-1.9) |
| **PD 5** | 10.2 (30/2946)  (7.1-14.5) | 5.4 (16/2946)  (3.3-8.8) | 4.8  (0.3-9.3) | 4.4% (131/2946)  (3.8-5.3) | 2.7% (81/2946)  (2.2-3.4) | 1.7  (0.8-2.7) | 5.5% (161/2946)  (4.7-6.3) | 3.3% (97/2946)  (2.7-4.0) | 2.2  (1.1-3.2) |
| **Overall** | 8.7 (128/14730)  (7.3-10.3) | 6.4 (95/14730)  (5.3-7.9) | 2.3  (0.3-4.3) | 2.7% (401/14730)  (2.5-3.0) | 1.8% (269/14730)  (1.6-2.1) | 0.9  (0.6-1.2) | 3.6% (529/14730)  (3.3-3.9) | 2.5% (364/14730)  (2.2-2.7) | 1.1  (0.7-1.5) |
|  |  |  |  |  |  |  |  |  |  |
| **DA 1** | 6.1 (18/2946)  (3.9-9.6) | 5.8 (17/2946)  (3.6-9.2) | 0.3  (-3.6-4.3) | 1.5% (45/2946)  (1.1-2.0) | 1.2% (34/2946)  (0.8-1.6) | 0.4  (-0.2-1.0) | 2.1% (63/2946)  (1.7-2.7) | 1.7% (51/2946)  (1.3-2.3) | 0.4  (-0.3-1.1) |
| **DA 2** | 7.8 (23/2946)  (5.2-11.7) | 7.1 (21/2946)  (4.7-10.9) | 0.7  (-3.7-5.1) | 1.9% (55/2946)  (1.4-2.4) | 1.4% (41/2946)  (1.0-1.9) | 0.5  (-0.2-1.1) | 2.6% (78/2946)  (2.1-3.3) | 2.1% (62/2946)  (1.6-2.7) | 0.5  (-0.2-1.3) |
| **DA 3** | 8.1 (24/2947)  (5.5-12.1) | 6.1 (18/2947)  (3.9-9.6) | 2.0  (-2.3-6.3) | 2.5% (75/2947)  (2.0-3.2) | 1.6% (48/2947)  (1.2-2.2) | 0.9  (0.2-1.7) | 3.4% (99/2947)  (2.8-4.1) | 2.2% (66/2947)  (1.8-2.8) | 1.1  (0.3-2.0) |
| **DA 4** | 11.2 (33/2945)  (8.0-15.7) | 7.5 (22/2945)  (4.9-11.3) | 3.7  (-1.2-8.6) | 3.3% (98/2945)  (2.7-4.0) | 1.9% (56/2945)  (1.5-2.5) | 1.4  (0.6-2.2) | 4.4% (131/2945)  (3.8-5.3) | 2.6% (78/2945)  (2.1-3.3) | 1.8  (0.9-2.7) |
| **DA 5** | 10.2 (30/2946)  (7.1-14.5) | 5.8 (17/2946)  (3.6-9.2) | 4.4  (-0.1-9.0) | 4.3% (128/2946)  (3.7-5.1) | 3.1% (90/2946)  (2.5-3.7) | 1.3  (0.3-2.3) | 5.4% (158/2946)  (4.6-6.2) | 3.6% (107/2946)  (3.0-4.4) | 1.7  (0.7-2.8) |
| **Overall** | 8.7 (128/14730)  (7.3-10.3) | 6.4 (95/14730)  (5.3-7.9) | 2.3  (0.3-4.3) | 2.7% (401/14730)  (2.5-3.0) | 1.8% (269/14730)  (1.6-2.1) | 0.9  (0.6-1.2) | 3.6% (529/14730)  (3.3-3.9) | 2.5% (364/14730)  (2.2-2.7) | 1.1  (0.7-1.5) |

CDR = cancer detection rate per 1000 women screened. DBT = digital breast tomosynthesis. CI = confidence interval. DM = digital mammography. FP = false positive. pp = percentage points. PD = breast percent density. DA = absolute dense area.

**Supplemental Table 3 Biopsy Rate, Positive Predictive Value for Recall, and Positive Predictive Value for Biopsy for Digital Breast Tomosynthesis and Digital Mammography in All Quintiles**

| **Quintile** | **Biopsy DBT**  **% (n)**  **(95% CI)** | **Biopsy DM**  **% (n)**  **(95% CI)** | **Difference Biopsy**  **(pp)**  **(95% CI)** | **PPV-1 DBT**  **% (n)**  **(95% CI)** | **PPV-1 DM**  **% (n)**  **(95% CI)** | **Difference PPV-1**  **(pp)**  **(95% CI)** | **PPV-3 DBT**  **% (n)**  **(95% CI)** | **PPV-3 DM**  **% (n)**  **(95% CI)** | **Difference PPV-3**  **(pp)**  **(95% CI)** |
| --- | --- | --- | --- | --- | --- | --- | --- | --- | --- |
| **PD 1** | 1.0% (30/2946)  (0.7-1.4) | 0.8% (25/2946)  (0.6-1.2) | 0.2  (-0.3-0.7) | 41.7% (20/48)  (28.8-55.7) | 43.2% (16/37)  (28.7-59.1) | -1.6  (-22.8-19.6) | 66.7% (20/30)  (48.8-80.8) | 64.0% (16/25)  (44.5-79.8) | 2.7  (-22.6-27.9) |
| **PD 2** | 1.6% (46/2946)  (1.2-2.1) | 1.3% (37/2946)  (0.9-1.7) | 0.3  (-0.3-0.9) | 26.1% (23/88)  (18.1-36.2) | 28.6% (18/63)  (18.9-40.7) | -2.4  (-16.8-12.0) | 50.0% (23/46)  (36.1-63.9) | 48.6% (18/37)  (33.4-64.1) | 1.4  (-20.3-23.0) |
| **PD 3** | 2.3% (67/2946)  (1.8-2.9) | 1.8% (53/2946)  (1.4-2.3) | 0.5  (-0.3-1.2) | 25.2% (29/115)  (18.2-33.9) | 31.6% (25/79)  (22.4-42.5) | -6.4  (-19.3-6.4) | 43.3% (29/67)  (32.1-55.2) | 47.2% (25/53)  (34.4-60.3) | -3.9  (-21.8-14.0) |
| **PD 4** | 2.2% (65/2946)  (1.7-2.8) | 1.6% (48/2946)  (1.2-2.2) | 0.6  (-0.1-1.3) | 22.2% (26/117)  (15.6-30.6) | 22.7% (20/88)  (15.2-32.5) | -0.5  (-12.0-11.0) | 40.0% (26/65)  (29.0-52.1) | 41.7% (20/48)  (28.8-55.7) | -1.7  (-20.0-16.7) |
| **PD 5** | 3.1% (91/2946)  (2.5-3.8) | 2.1% (62/2946)  (1.6-2.7) | 1.0  (0.2-1.8) | 18.6% (30/161)  (13.4-25.4) | 16.5% (16/97)  (10.4-25.1) | 2.1  (-7.5-11.8) | 33.0% (30/91)  (24.2-43.1) | 25.8% (16/62)  (16.6-37.9) | 7.2  (-7.6-22.0) |
| **Overall** | 2.0% (299/14730)  (1.8-2.3) | 1.5% (225/14730)  (1.3-1.7) | 0.5  (0.2-0.8) | 24.2% (128/529)  (20.7-28.0) | 26.1% (95/364)  (21.9-30.8) | -1.9  (-7.7-3.9) | 42.8% (128/299)  (37.3-48.5) | 42.2% (95/225)  (36.0-48.8) | 0.6  (-8.0-9.1) |
|  |  |  |  |  |  |  |  |  |  |
| **DA 1** | 1.1% (32/2946)  (0.8-1.5) | 1.0% (30/2946)  (0.7-1.4) | 0.1  (-0.5-0.6) | 28.6% (18/63)  (18.9-40.7) | 33.3% (17/51)  (22.0-47.0) | -4.8  (-21.8-12.3) | 56.3% (18/32)  (39.3-71.8) | 56.7% (17/30)  (39.2-72.6) | -0.4  (-25.1-24.3) |
| **DA 2** | 1.7% (49/2946)  (1.3-2.2) | 1.4% (42/2946)  (1.1-1.9) | 0.2  (-0.4-0.9) | 29.5% (23/78)  (20.5-40.4) | 33.9% (21/62)  (23.3-46.3) | -4.4  (-19.9-11.1) | 46.9% (23/49)  (33.7-60.6) | 50.0% (21/42)  (35.5-64.5) | -3.1  (-23.7-17.5) |
| **DA 3** | 1.8% (52/2947)  (1.3-2.3) | 1.3% (38/2947)  (0.9-1.8) | 0.5  (-0.2-1.1) | 24.2% (24/99)  (16.9-33.5) | 27.3% (18/66)  (18.0-39.0) | -3.0  (-16.6-10.5) | 46.2% (24/52)  (33.3-59.5) | 47.4% (18/38)  (32.5-62.7) | -1.2  (-22.1-19.7) |
| **DA 4** | 2.6% (76/2945)  (2.1-3.2) | 1.7% (49/2945)  (1.3-2.2) | 0.9  (0.2-1.7) | 25.2% (33/131)  (18.5-33.3) | 28.2% (22/78)  (19.4-39.0) | -3.0  (-15.4-9.3) | 43.4% (33/76)  (32.9-54.6) | 44.9% (22/49)  (31.9-58.7) | -1.5  (-19.3-16.4) |
| **DA 5** | 3.1% (90/2946)  (2.5-3.7) | 2.2% (66/2946)  (1.8-2.8) | 0.8  (0.0-1.6) | 19.0% (30/158)  (13.6-25.8) | 15.9% (17/107)  (10.2-24.0) | 3.1  (-6.3-12.5) | 33.3% (30/90)  (24.5-43.6) | 25.8% (17/66)  (16.7-37.4) | 7.6  (-7.0-22.2) |
| **Overall** | 2.0% (299/14730)  (1.8-2.3) | 1.5% (225/14730)  (1.3-1.7) | 0.5  (0.2-0.8) | 24.2% (128/529)  (20.7-28.0) | 26.1% (95/364)  (21.9-30.8) | -1.9  (-7.7-3.9) | 42.8% (128/299)  (37.3-48.5) | 42.2% (95/225)  (36.0-48.8) | 0.6  (-8.0-9.1) |

DBT = digital breast tomosynthesis. CI = confidence interval. DM = digital mammography. pp = percentage points. PPV-1 = positive predictive value of recall. PPV-3 = positive predictive value of biopsy. PD = breast percent density. DA = absolute dense area.

| **Quintile of women 40–49 years old** | **Women (n)** | **PD (%)** | **BI-RADS Density (mean)** | **Age (mean)** | **No Previous Screening n (%)** | **Cancer and IC n (%)** | **FP n (%)** |
| --- | --- | --- | --- | --- | --- | --- | --- |
| **PD 1^40-49^** | 826 | < 18.95% | 1.77 | 46 | 177/826 (21.4%) | 8/826 (1.0%) | 27/826 (3.3%) |
| **PD 2^40-49^** | 826 | 18.95–29.14% | 2.41 | 45 | 217/826 (26.3%) | 4/826 (0.5%) | 42/826 (5.1%) |
| **PD 3^40-49^** | 826 | 29.14–42.00% | 2.83 | 45 | 232/826 (28.1%) | 3/826 (0.4%) | 51/826 (6.2%) |
| **PD 4^40-49^** | 826 | 42.00–55.36% | 3.16 | 45 | 213/826 (25.8%) | 8/826 (1.0%) | 51/826 (6.2%) |
| **PD 5^40-49^** | 826 | > 55.36% | 3.48 | 44 | 229/826 (27.7%) | 5/826 (0.6%) | 50/826 (6.1%) |

**Supplemental Table 4a** **Descriptive Statistics of Breast Percent Density Quintiles^40-49^ for Women 40–49 Years Old**

**a**

**Supplemental Table 4b** **Descriptive Statistics of Absolute Dense Area Quintiles^40-49^ for Women 40–49 Years Old**

| **Quintile of women 40–49 years old** | **Women (n)** | **DA (cm^2^)** | **BI-RADS Density (mean)** | **Age (mean)** | **No Previous Screening n (%)** | **Cancer and IC n (%)** | **FP n (%)** |
| --- | --- | --- | --- | --- | --- | --- | --- |
| **DA 1^40-49^** | 826 | < 29.94 cm^2^ | 2.05 | 46 | 210/826 (25.4%) | 2/826 (0.2%) | 33/826 (4.0%) |
| **DA 2^40-49^** | 826 | 29.94–39.14 cm^2^ | 2.48 | 45 | 192/826 (23.2%) | 6/826 (0.7%) | 36/826 (4.4%) |
| **DA 3^40-49^** | 826 | 39.14–49.24 cm^2^ | 2.82 | 45 | 221/826 (26.8%) | 8/826 (1.0%) | 43/826 (5.2%) |
| **DA 4^40-49^** | 826 | 49.24–63.94 cm^2^ | 3.04 | 45 | 225/826 (27.2%) | 5/826 (0.6%) | 51/826 (6.2%) |
| **DA 5^40-49^** | 826 | > 63.94 cm^2^ | 3.29 | 45 | 220/826 (26.6%) | 7/826 (0.8%) | 58/826 (7.0%) |

**b**

PD = breast percent density. BI-RADS = Breast Imaging Reporting and Data System 4^th^ ed. IC = interval cancer. FP = false positive. DA = absolute dense area.

**Supplemental Table 5 Sensitivity, Specificity, and Cancer Detection Rate among Women 40–49 Years Old in All BI-RADS Density Categories**

| **BI-RADS Density Category of Women 40–49** | **Sensitivity DBT**  **% (n) (95% CI)** | **Sensitivity DM**  **% (n) (95% CI)** | **Specificity DBT**  **% (n) (95% CI)** | **Specificity DM**  **% (n) (95% CI)** | **CDR DBT**  **(n) (95% CI)** | **CDR DM**  **(n) (95% CI)** |
| --- | --- | --- | --- | --- | --- | --- |
| **BI-RADS 1** | 100.0% (1/1)  (20.7-100.0) | 100.0% (1/1)  (20.7-100.0) | 99.0% (308/311)  (97.2-99.7) | 99.4% (309/311)  (97.7-99.8) | 3.2 (1/312)  (0.6-17.9) | 3.2 (1/312)  (0.6-17.9) |
| **BI-RADS 2** | 87.5% (7/8)  (52.9-97.8) | 50.0% (4/8)  (21.5-78.5) | 96.4% (1059/1099)  (95.1-97.3) | 97.3% (1069/1099)  (96.1-98.1) | 6.3 (7/1107)  (3.1-13.0) | 3.6 (4/1107)  (1.4-9.3) |
| **BI-RADS 3** | 72.7% (8/11)  (43.4-90.3) | 45.5% (5/11)  (21.3-72.0) | 94.9% (1661/1750)  (93.8-95.8) | 96.4% (1687/1750)  (95.4-97.2) | 4.5 (8/1761)  (2.3-8.9) | 2.8 (5/1761)  (1.2-6.6) |
| **BI-RADS 4** | 87.5% (7/8)  (52.9-97.8) | 62.5% (5/8)  (30.6-86.3) | 94.3% (649/688)  (92.3-95.8) | 95.6% (658/688)  (93.8-96.9) | 10.1 (7/696)  (4.9-20.6) | 7.2 (5/696)  (3.1-16.7) |
| **Overall, with valid BI-RADS Density** | 82.1% (23/28)  (64.4-92.1) | 53.6% (15/28)  (35.8-70.5) | 95.6% (3677/3848)  (94.9-96.2) | 96.8% (3723/3848)  (96.1-97.3) | 5.9 (23/3876)  (4.0-8.9) | 3.9 (15/3876)  (2.3-6.4) |

BI-RADS = Breast Imaging Reporting and Data System 4^th^ ed. DBT = digital breast tomosynthesis. CI = confidence interval. DM = digital mammography. CDR = cancer detection rate per 1000 women screened.

| **BI-RADS Density Category** | **Sensitivity DBT**  **% (n) (95% CI)** | **Sensitivity DM**  **% (n) (95% CI)** | **P Value** | **Specificity DBT**  **% (n) (95% CI)** | **Specificity DM**  **% (n) (95% CI)** | **P Value** |
| --- | --- | --- | --- | --- | --- | --- |
| **BI-RADS 1** | 90.9% (10/11)  (62.3-98.4) | 54.5% (6/11)  (28.0-78.7) | .13 | 98.7% (2272/2302)  (98.1- 99.1) | 99.5% (2290/2302)  (99.1-99.7) | .001 |
| **BI-RADS 2** | 78.4% (40/51)  (65.4-87.5) | 56.9% (29/51)  (43.3-69.5) | .013 | 97.8% (5179/5296)  (97.4-98.2) | 98.4% (5211/5296)  (98.0-98.7) | .003 |
| **BI-RADS 3** | 78.4% (58/74)  (67.7-86.2) | 66.2% (49/74)  (54.9-76.0) | .06 | 96.1% (4650/4837)  (95.6-96.6) | 97.4% (4710/4837)  (96.9-97.8) | < .001 |
| **BI-RADS 4** | 90.9% (20/22)  (72.2-97.5) | 50.0% (11/22)  (30.7-69.3) | .004 | 94.5% (1142/1208)  (93.1-95.7) | 96.3% (1163/1208)  (95.1-97.2) | .008 |
| **Overall, with valid BI-RADS Density** | 81.0% (129/159)  (74.2-86.4) | 60.1% (95/158)  (52.3-67.4) | < .001 | 97.1% (13243/13643)  (96.8-97.3) | 98.0% (13374/13643)  (97.8-98.2) | < .001 |

**Supplemental Table 6 Sensitivity and Specificity of Digital Breast Tomosynthesis and Digital Mammography in All BI-RADS Density Categories**

BI-RADS = Breast Imaging Reporting and Data System 4^th^ ed. DBT = digital breast tomosynthesis. CI = confidence interval. DM = digital mammography.

**Supplemental Table 7 Cancer Detection Rate and False Positives for Digital Breast Tomosynthesis and Digital Mammography** **in All BI-RADS Density Categories**

| **BI-RADS Density Category** | **CDR DBT**  **(n) (95% CI)** | **CDR DM**  **(n) (95% CI)** | **Difference CDR (95% CI)** | **FP DBT**  **% (n) (95% CI)** | **FP DM**  **% (n) (95% CI)** | **Difference FP (pp) (95% CI)** |
| --- | --- | --- | --- | --- | --- | --- |
| **BI-RADS 1** | 4.3 (10/2313)  (2.4-7.9) | 2.6 (6/2313)  (1.2-5.6) | 1.7  (-1.7-5.1) | 1.3% (30/2313)  (0.9-1.8) | 0.5% (12/2313)  (0.3-0.9) | 0.8  (0.2-1.3) |
| **BI-RADS 2** | 7.5 (40/5347)  (5.5-10.2) | 5.4 (29/5347)  (3.8-7.8) | 2.1  (-0.9-5.1) | 2.2% (117/5347)  (1.8-2.6) | 1.6% (85/5347)  (1.3-2.0) | 0.6  (0.1-1.1) |
| **BI-RADS 3** | 11.8 (58/4911)  (9.1-15.2) | 10.0 (49/4911)  (7.6-13.2) | 1.8  (-2.3-5.9) | 3.8% (188/4911)  (3.3-4.4) | 2.6% (127/4911)  (2.2-3.1) | 1.2  (0.6-1.9) |
| **BI-RADS 4** | 16.3 (20/1230)  (10.6-25.0) | 8.9 (11/1230)  (5.0-15.9) | 7.4  (-1.4-16.2) | 5.4% (66/1230)  (4.2-6.8) | 3.7% (45/1230)  (2.7-4.9) | 1.7  (0.1-3.4) |
| **Overall, with valid BI-RADS Density** | 9.3 (128/13801)  (7.8-11.0) | 6.9 (95/13801)  (5.7-8.5) | 2.4  (0.3-4.5) | 2.9% (401/13801)  (2.6-3.2) | 1.9% (269/13801)  (1.7-2.2) | 1.0  (0.6-1.3) |

BI-RADS = Breast Imaging Reporting and Data System 4^th^ ed. CDR = cancer detection rate per 1000 women screened. DBT = digital breast tomosynthesis. CI = confidence interval. DM = digital mammography. FP = false positive. pp = percentage points.

| **BI-RADS Density Category** | **Biopsy DBT**  **% (n) (95% CI)** | **Biopsy DM**  **% (n) (95% CI)** | **Difference Biopsy (pp)**  **(95% CI)** | **Recall DBT**  **% (n) (95% CI)** | **Recall DM**  **% (n) (95% CI)** | **Difference Recall (pp)**  **(95% CI)** |
| --- | --- | --- | --- | --- | --- | --- |
| **BI-RADS 1** | 0.9% (20/2313)  (0.6-1.3) | 0.5% (12/2313)  (0.3-0.9) | 0.3  (-0.1-0.8) | 1.7% (40/2313)  (1.3-2.3) | 0.8% (18/2313)  (0.5-1.2) | 1.0  (0.3-1.6) |
| **BI-RADS 2** | 1.6% (83/5347)  (1.3-1.9) | 1.2% (62/5347)  (0.9-1.5) | 0.4  (-0.1-0.8) | 2.9% (157/5347)  (2.5-3.4) | 2.1% (114/5347)  (1.8-2.6) | 0.8  (0.2-1.4) |
| **BI-RADS 3** | 2.9% 143/4911)  (2.5-3.4) | 2.4% (117/4911)  (2.0-2.8) | 0.5  (-0.1-1.2) | 5.0% (246/4911)  (4.4-5.7) | 3.6% (176/4911)  (3.1-4.1) | 1.4  (0.6-2.2) |
| **BI-RADS 4** | 4.3% (53/1230)  (3.3-5.6) | 2.8% (34/1230)  (2.0-3.8) | 1.5  (0.1-3.0) | 7.0% (86/1230)  (5.7-8.6) | 4.6% (56/1230)  (3.5-5.9) | 2.4  (0.6-4.3) |
| **Overall, with valid BI-RADS Density** | 2.2% (299/13801)  (1.9-2.4) | 1.6% (225/13801)  (1.4-1.9) | 0.5  (0.2-0.9) | 3.8% (529/13801)  (3.5-4.2) | 2.6% (364/13801)  (2.4-2.9) | 1.2  (0.8-1.6) |

**Supplemental Table 8 Biopsy and Recall Rates for Digital Breast Tomosynthesis and Digital Mammography** **in All BI-RADS Density Categories**

BI-RADS = Breast Imaging Reporting and Data System 4^th^ ed. DBT = digital breast tomosynthesis. CI = confidence interval. DM = digital mammography. pp = percentage points.

| **BI-RADS Density Category** | **PPV-1 DBT**  **% (n) (95% CI)** | **PPV-1 DM**  **% (n) (95% CI)** | **Difference PPV-1 (pp)**  **(95 % CI)** | **PPV-3 DBT**  **% (n) (95% CI)** | **PPV-3 DM**  **% (n) (95% CI)** | **Difference PPV-3 (pp)**  **(95 % CI)** |
| --- | --- | --- | --- | --- | --- | --- |
| **BI-RADS 1** | 25.0% (10/40)  (14.2-40.2) | 33.3% (6/18)  (16.3-56.3) | -8.3  (-33.2-16.5) | 50.0% (10/20) (29.9-70.1) | 50.0% (6/12)  (25.4-74.6) | 0.0  (-35.8-35.8) |
| **BI-RADS 2** | 25.5% (40/157) (19.3-32.8) | 25.4% (29/114)  (18.3-34.1) | 0.0  (-10.5-10.6) | 48.2% (40/83) (37.8-58.8) | 46.8% (29/62)  (34.9-59.0) | 1.4  (-15.0-17.9) |
| **BI-RADS 3** | 23.6% (58/246) (18.7-29.3) | 27.8% (49/176)  (21.7-34.9) | -4.3  (-12.7-4.2) | 40.6% (58/143) (32.9-48.8) | 41.9% (49/117)  (33.3-50.9) | -1.3  (-13.3-10.7) |
| **BI-RADS 4** | 23.3% (20/86) (15.6-33.2) | 19.6% (11/56)  (11.3-31.8) | 3.6  (-10.3-17.5) | 37.7% (20/53) (25.9-51.2) | 32.4% (11/34)  (19.1-49.2) | 5.4  (-15.2-26.0) |
| **Overall, with valid BI-RADS Density** | 24.2% (128/529)  (20.7-28.0) | 26.1% (95/364)  (21.9-30.8) | -1.9  (-7.7-3.9) | 42.8% (128/299)  (37.3-48.5) | 42.2% (95/225)  (36.0-48.8) | 0.6  (-8.0-9.1) |

**Supplemental Table 9 Positive Predictive Values of Recall and Biopsy in Digital Breast Tomosynthesis and Digital Mammography in All BI-RADS Density Categories**

BI-RADS = Breast Imaging Reporting and Data System 4^th^ ed. PPV-1 = positive predictive value of recall. DBT = digital breast tomosynthesis. CI = confidence interval. DM = digital mammography. pp = percentage points. PPV-3 = positive predictive value of biopsy.
